# Evaluation of the learning curve in robot‐assisted knee arthroplasty: A Systematic review

**DOI:** 10.1002/jeo2.70292

**Published:** 2025-07-13

**Authors:** Emidio Di Gialleonardo, Guido Bocchino, Giacomo Capece, Matteo Salvini, Marco Barbaliscia, Giuseppe Malerba, Omar El Ezzo, Giulio Maccauro, Raffaele Vitiello

**Affiliations:** ^1^ Department of Orthopedics and Geriatric Sciences Catholic University of the Sacred Heart Rome Italy; ^2^ Department of Orthopedics, Ageing and Rheumatological Sciences Fondazione Policlinico Universitario A. Gemelli IRCCS Rome Italy

**Keywords:** arthroplasty, knee, learning curve, robotic, TKA

## Abstract

**Purpose:**

Robot‐assisted (RA) surgery has transformed total knee arthroplasties (TKA) and unicompartmental knee arthroplasties (UKA) by significantly enhancing the accuracy of prosthetic implantation and reducing complications. Different robotic systems offer unique approaches to assist surgeons during procedures.

**Methods:**

This systematic review adhered to Preferred Reporting Items for Systematic Reviews and Meta‐Analyses (PRISMA) guidelines and evaluated the learning curve associated with RA technologies, focusing on their surgical efficacy. A comprehensive literature search identified and analysed studies published since 2010, ultimately selecting 28 relevant articles that addressed robotic techniques in knee arthroplasty.

**Results:**

The findings indicate that surgeons generally require an average of 21 procedures to achieve proficiency with robotic techniques. However, the learning curve varies among robotic systems: MAKO requires 15–25 cases, ROSA 20–30 cases and NAVIO 18–28 cases for surgeons to reach proficiency. The MAKO system emerged as the most frequently used (33.3% of studies), followed by ROSA (23.3%) and NAVIO (16.7%). Evidence suggests that the adoption of these robotic systems is associated with reduced operative times and lower rates of postoperative complications, thereby improving overall surgical outcomes.

**Conclusions:**

RA arthroplasties present significant advancements in surgical precision and patient outcomes. With targeted investments in training and technology, the adoption of robotic techniques could further increase, ultimately enhancing the quality of orthopaedic care and patient recovery. This review highlights the importance of addressing training needs and resource allocation to fully realise the potential of robotic surgery in knee arthroplasty.

**Level of Evidence:**

Not applicable, systematic review.

**Abbreviations**
CR: cruciate retaining
cTKA: conventional total knee arthroplasties
cUKA: conventional unicompartmental knee arthroplasties
PS: posteriorly stabilised
RA: robot‐assisted
Ra‐TKA: robot‐assisted total knee arthroplasties
Ra‐UKA: robot‐assisted unicompartmental knee arthroplasties
RIAP: Italian Arthroplasty Registry
TKA: total knee arthroplasties
UKA: unicompartmental knee arthroplasties

## INTRODUCTION

In recent years, robot‐assisted (RA) surgery has revolutionised total knee arthroplasties (TKA) and unicompartmental knee arthroplasties (UKA), improving the accuracy of prosthetic implantation and reducing the risk of complications [16]. As the adoption of these technologies grows, understanding the learning curve associated with RA procedures is crucial for optimising surgical training, ensuring cost‐effectiveness and maximising patient outcomes. The ability of surgeons to rapidly achieve proficiency with these systems directly impacts procedural efficiency, complication rates and long‐term implant success, influencing both healthcare expenditures and the accessibility of robotic surgery on a broader scale. The main systems driving this innovation include NAVIO (Smith & Nephew S.r.l, MI), MAKO (Stryker Way Portage, MI Stati Uniti) and ROSA (Zimmer Biomet, Warsaw, IN, USA). These systems use different approaches to assist the surgeon during surgery. NAVIO operates without preoperative imaging, relying instead on intraoperative sensors to create a real‐time 3D model of the patient's anatomy. Unlike other systems, NAVIO does not feature a robotic arm; instead, the surgeon manually performs bone cuts while receiving real‐time navigation feedback to optimise prosthesis placement [3]. In contrast, MAKO uses a preoperative CT scan to generate a detailed 3D model of the patient's knee or hip, allowing precise planning before the operation. During surgery, MAKO features a robotic arm that guides the surgeon's bone‐cutting movements according to the preoperative plan based on the images [23, 26]. MAKO has the advantage of being used for both knee and hip arthroplasties, extending its application to hip prosthetic surgeries. Like MAKO, robotic surgical assistant (ROSA) is used for both knee and hip surgeries, combining preoperative imaging such as CT scans or X‐rays with intraoperative sensors that provide real‐time updates on the patient's anatomy [28]. ROSA also features a robotic arm that guides the surgeon in making bone cuts and positioning the prosthesis [7, 8]. A significant advantage common to these robotic systems is the ability to assess prosthetic implant stability during surgery. NAVIO, through its sensors, provides real‐time evaluation of ligament balance and implant stability. Similarly, MAKO and ROSA offer intraoperative feedback on the stability and alignment of the prosthesis, allowing the surgeon to make precise adjustments before finalising the implant. This real‐time control significantly improves the quality of the surgery, reducing the risk of postoperative complications and enhancing patient recovery [17]. These features help surgeons refine component alignment and ensure optimal biomechanical function, which is particularly beneficial during the learning phase. As RA procedures become more prevalent, the duration of the learning curve and the factors influencing surgical proficiency—such as case volume, prior experience with robotic platforms and institutional support—become critical areas of investigation.

National Italian data, as reported by the RIAP (Italian Arthroplasty Registry) project, show a significant increase in TKA from 2001 to 2015 [30]. In the last year alone, approximately 65,000 knee prostheses and 60,000 hip prostheses were performed, confirming steady growth over the years. However, internationally, the trend is even more pronounced. In the United States, around 700,000 total knee replacements are performed each year, with projections exceeding 3.5 million annual procedures by 2030, driven by an aging population and rising osteoarthritis incidence [42]. This includes both conventional manual techniques (cTKA or cUKA) and robot‐assisted (Ra‐TKA or Ra‐UKA), although robotic surgery still represents a smaller, but steadily growing, portion of these procedures. Similarly, European countries like the United Kingdom and Germany are seeing a similar rise in knee prosthesis surgeries, contributing to the growing adoption of robotic technologies. The use of robotic surgery is steadily increasing worldwide. In the United States, the adoption of robotic technologies for knee replacement surgeries has grown more than sixfold since 2017. Studies show that robotic systems like MAKO, NAVIO and ROSA not only improve the accuracy of prosthesis placement but also reduce the risk of postoperative complications and allow for faster recovery compared with traditional techniques [6]. The growing demand for robotic surgical interventions is driven by the ability of these systems to improve clinical outcomes and optimise operating times, particularly in complex cases. In addition to these three main systems, other robotic devices are emerging in the orthopaedic field [29].

For instance, OMNIBotics by Corin (Corin Italia SRL,Fagagna, Italy) uses intraoperative sensors to perform precise bone cuts and balance ligaments without relying on preoperative imaging. TSolution One by THINK Surgical (THINK Surgical, Inc.) uses preoperative imaging to plan the surgery and a robotic arm to make bone cuts [18]. CORI Surgical System, an advanced version of NAVIO by Smith & Nephew, operates similarly without requiring preoperative imaging, using intraoperative sensors to create a realtime 3D model [4]. Finally, Velys by DePuy Synthes (Johnson & Johnson Medical SpA) integrates both preoperative and intraoperative imaging systems to optimise surgical accuracy, with a robotic arm guiding the precise placement of the prosthesis. Each of these systems presents unique challenges in terms of surgeon adaptation, influencing how quickly proficiency can be achieved.

This review aims to analyse the learning curve associated with robotic knee arthroplasties, identifying the critical threshold at which a surgeon can be considered autonomous and proficient in their use. By evaluating complication rates, operating time optimisation and the number of procedures required for competency, we seek to provide insight into the adoption of robotic systems in surgical practice. Understanding these factors is essential for refining training programs, justifying the economic investment in robotic platforms, and ultimately improving patient outcomes.

## MATERIALS AND METHODS

The review adhered to the Preferred Reporting Items for Systematic Reviews and Meta‐Analyses (PRISMA) guidelines [24], ensuring a comprehensive and systematic approach to data retrieval and synthesis. This systematic review has been appropriately registered with the International Prospective Register of Systematic Reviews (PROSPERO).

### Search strategy

**Figure 1:**
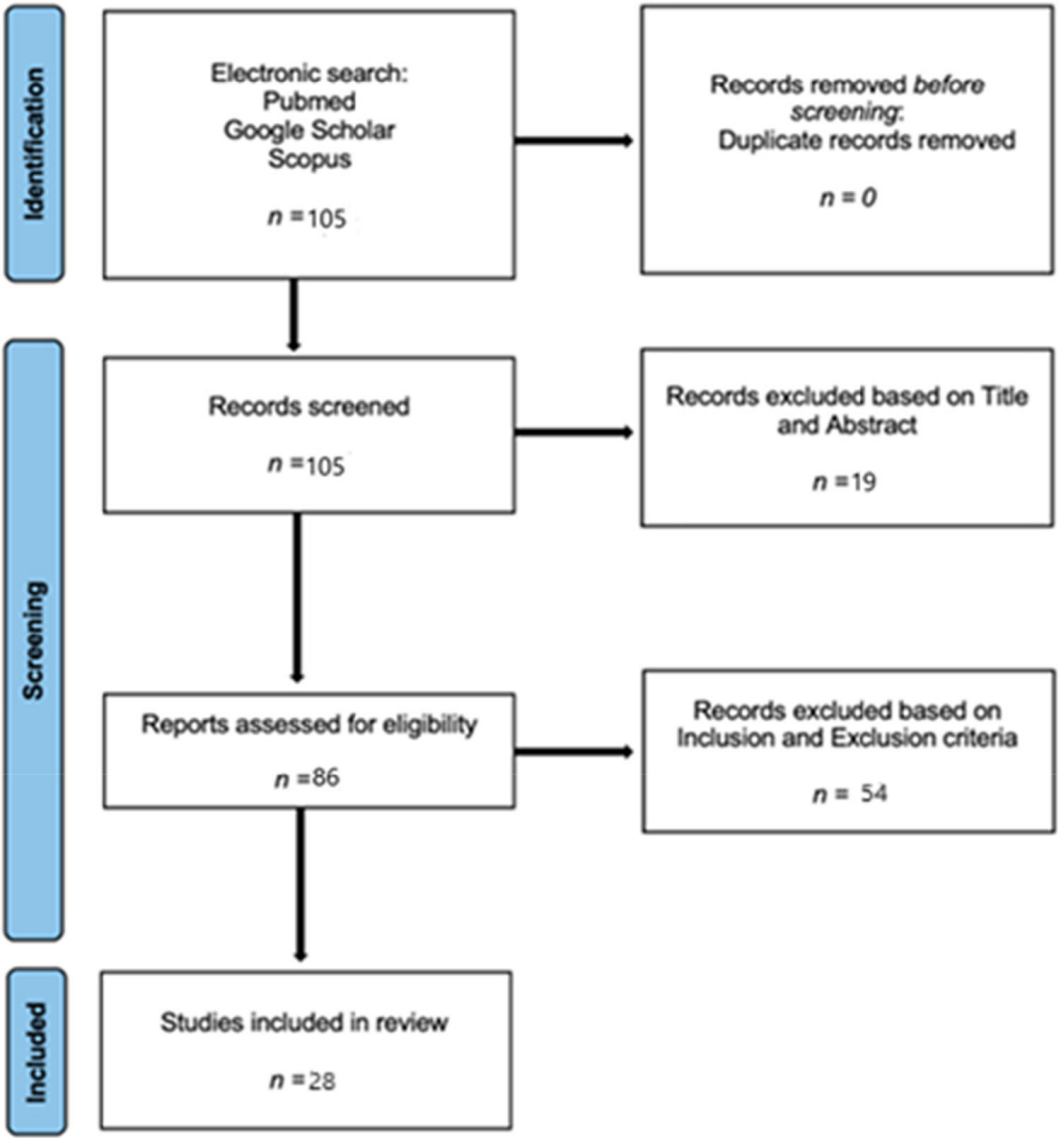
PRISMA flow chart. PRISMA, Preferred Reporting Items for Systematic Reviews and Meta‐Analyses.

A systematic review was conducted in accordance with the PRISMA guidelines (Figure 1), up to January 2024. The literature search focused on studies investigating the learning curve in Ra‐TKA or Ra‐UKA and its impact on the reduction of complications and operative times. The search was conducted across PubMed, Scopus and Web of Science due to their comprehensive coverage of orthopaedic literature. While databases such as Embase and Cochrane were not included, future systematic reviews may benefit from broader database selection. The following search string was applied to PubMed: (robotic) AND (knee) AND (learning curve). This search yielded a total of 105 articles. After an initial screening based on titles and abstract, 86 articles were selected. Further abstract analysis determined the eligibility of 43 articles. Finally, after a comprehensive full‐text review, 28 studies were selected as relevant to our research. The search was limited to studies published from 2010 to the present, in the English language. Reference lists of selected studies were also reviewed to identify any potentially relevant papers that were missed in the initial search.

### Inclusion and exclusion criteria

This review considers studies involving patients undergoing RA total or unicompartmental knee arthroplasty (TKA/UKA) using technologies such as MAKO, NAVIO, ROSA and other similar robotic systems. The inclusion criteria require that the studies be clinical, prospective or retrospective, and evaluate the surgeons’ learning curve through measures such as operative times, implant positioning accuracy and postoperative complications. Only studies published from 2010 onward, in English, are included, as they reflect the most current robotic technologies and surgical techniques, ensuring relevance to present‐day clinical practice. While earlier studies may offer historical insights, the rapid evolution of robotic systems in the past decade has significantly changed their functionality, making older data less applicable to contemporary learning curves (Table 1).

**Table 1:** Inclusion and exclusion criteria.

	Inclusion	Exclusion
Population	Patients aged > 18 years undergoing robot‐assisted total or unicompartmental knee arthroplasty (TKA/UKA).	Patients aged < 18 years
		Fewer than five cases
Intervention	Robotic surgery (TKA/UKA) with systems such as MAKO, NAVIO, ROSA, Cori and so on.	Only conventional surgery (TKA/UKA)
Design	Prospective, retrospective or cohort clinical studies that evaluate the learning curve and effectiveness of robotic surgery.	Case report, Case series, Reviews, editorials.
Other	Published from 2010 onwards, in English, with quantitative data on the learning curve (operating times, implant accuracy, complications).	Studies not directly related to the learning curve in robotic‐assisted knee arthroplasty

Abbreviations: ROSA, robotic surgical assistant; TKA, total knee arthroplasties; UKA, unicompartmental knee arthroplasties.

Studies that do not specifically address knee surgery or the use of robotic systems, as well as those that do not assess the learning curve and studies not directly related to the learning curve in RA knee arthroplasty, are excluded. Other excluded studies include reviews, editorials, articles with insufficient data, studies with very small samples (fewer than five cases) or works published before 2010.

### Data extraction and analysis

Four independent reviewers screened titles and abstracts, with discrepancies resolved through discussion and consensus. If agreement was not reached, a senior reviewer made the final decision. Full‐text articles were obtained for any abstracts that met the inclusion criteria or where there was uncertainty. Data extraction focused on participant demographics, risk factors, surgical outcomes, operative times and complication rates. Each study's methodological quality was assessed using the Methodological Index for Non‐Randomised Studies (MINORS) score, which yields a maximum score of 16 for noncomparative studies and 24 for comparative studies [32]. The mean value of the MINORS score among the various studies analysed was 15.2 (range 10–24). Most of the studies had a score between 14 and 17. Two authors independently assigned the MINORS score, with consensus reached on the final score.

Data concerning the learning curve, operative times and complication rates were extracted, reviewed and compiled for further analysis. Statistical significance was defined as *p* < 0.05. SPSS software (SPSS, Inc.) was used to tabulate the collected data. Prior to statistical comparisons, data were assessed for normality using the Shapiro–Wilk test. One‐way analysis of variance (ANOVA) was used for normally distributed data, while the Kruskal–Wallis test with post hoc Bonferroni correction was applied for non‐normally distributed variables. Categorical variables are presented as frequencies and percentages, and continuous variables are reported as means with standard deviations. All numerical data were rounded to one decimal place for precision.

## RESULTS

### Demographics data

The number of patients undergoing total or single‐compartment knee replacement surgery was 4664. The total number of patients undergoing total or mono‐compartmental knee arthroplasty surgery by robotic technique was 3950 (84.7%). The number of surgeons included in the study was 193. In four studies (14,3%), it was not specified how many surgeons were analysed (Table 2).

**Table 2:** Demographic data.

First author	Number of cases	Number of surgeons	Surgeon's initial experience	Type of robot	Type of implant
Sodhi et al. [33]	240	2	Experienced	Stryker MAKO	TKA N/A
Kayani et al. [13]	120 (60 Ra‐TKA)	1	Experienced	Stryker MAKO	UKA
Kayani et al. [14]	120 (60 Ra‐TKA)	1	Experienced	Stryker MAKO	TKA PS
Naziri et al. [22]	80 (40 Ra‐TKA)	1	Experienced	Stryker MAKO	TKA N/A
Marchand et al. [20]	200 (140 Ra‐TKA)	1	Experienced	Stryker MAKO	TKA CR
Mahure et al. [19]	115	4	Experienced	THINK	72 TKA PS 43 TKA CR
Bell et al. [2]	60	1	Experienced	Smith & Nephew NAVIO	TKA PS
Savov et al. [31]	140 (70 Ra‐TKA)	1	Experienced	Smith & Nephew NAVIO	TKA PS
Probst et al. [27]	351	N/A	Not Experienced	N/A	TKA N/A
Vermue et al. [40]	30	1	Experienced	OMNIBotics	TKA N/A
Mei Lin Tay et al. [35]	101	3	Experienced	Stryker MAKO	TKA CS
Morrisey et al. [21]	165 (66 Ra‐TKA)	1	Experienced	VELYS™ DePuy Synthes	TKA CR
Chen et al. [9]	N/A	146	N/A	Various	TKA N/A
Thiengwittayaporn et al. [36]	152 (75 Ra‐TKA)	N/A	Experienced	Smith & Nephew NAVIO	TKA PS
Vanlommel et al. [38]	180 (90 Ra‐TKA)	3	Experienced	Zimmer Biomet ROSA	TKA PS
Stegelmann et al. [34]	200	2	Not Experienced	Smith & Nephew NAVIO	TKA PS
Ali et al. [1]	120	2	Experienced	Stryker MAKO	TKA N/A
Patel et al. [25]	604	1	Experienced	Stryker MAKO	TKA and UKA
Vaidya et al. [39]	100 (75 Ra‐TKA)	N/A	N/A	Smith & Nephew NAVIO	TKA
Bolam et al. [5]	136 (53 Ra‐TKA)	3	Experienced	Zimmer Biomet ROSA	TKA N/A
Knenanidis et al. [15]	200	N/A	Experienced	Zimmer Biomet ROSA	TKA PS
Jung et al. [12]	100 (50 Ra‐TKA)	1	Experienced	Stryker MAKO	TKA PS
Thongpulsawad et al. [37]	110	3	Experienced	ROSA Zimmer Biomet	TKA PS
Weaver et al. [41]	500	1	N/A	Smith & Nephew CORI	TKA N/A
Neira et al. [23]	90	3	1 Experienced and 2 Not Experienced	Zimmer Biomet ROSA	TKA PS
Ejnisman et al. [11]	321	5	Experienced and Not Experienced	ROSA Zimmer Biomet and MAKO Stryker	TKA PS
Zhang et al. [44]	90	3	Experienced	HURWA	TKA PS
Dragosloveanu et al. [10]	39	3	Experienced	Zimmer Biomet ROSA	TKA PS

Abbreviations: CR, cruciate retaining; PS, posteriorly stabilised; RA, robot‐assisted; TKA, total knee arthroplasties; UKA, unicompartmental knee arthroplasties.

### Type of robot and type of implant

The utilisation of robotic systems for knee arthroplasty was reported in 26 studies, accounting for 92.9% of the total. In two studies, the type of robot used was not specified (7.2%). The most commonly used type of robot was the Mako (Stryker) in 35.7% of cases (10/28). The second most commonly used type of robot was the Rosa (Zimmer Biomet), 25% of cases (7/28). The third most used type of robot was the Navio (Smith Nephew) in 17.9% of cases (5/28). Other robots used were: Velys (Depuy Synthes) in one study (3.6%), Cori (Smith Nephew) in one study (3.6%), Hurwa in one study (3.6%), TSolution One Total Knee Application (THINK Surgical) in one study (3.6%), OmniBotics in one study (3.6%). In 26 out of 28 studies analysed, the surgery consisted of total knee replacements, whereas only two studies analysed single‐compartment knee replacements. Posteriorly stabilised (PS) knee implants were implanted in 13 studies and cruciate retaining (CR) knee implants were implanted in three studies. Both PS and CR prostheses were analysed in a study by Siddhart et al. In 11 studies, it was not specified whether the total knee prosthesis was PS or CR (Table 2).

### Learning curve and surgeons experience

The number of cases needed to learn the robotic technique has been specified in 25 articles. In three articles, the number of cases needed was not specified. The average number of procedures required to achieve proficiency was 20.52. However, a notable variability was observed among studies, with reported learning curves ranging from as few as three cases to as many as 53 cases. This broad range suggests that multiple factors influence the learning process, including the surgeon's prior experience, the complexity of the robotic system, and the extent of institutional training support available. When comparing robotic platforms, variability in learning time was also observed. MAKO required 15–25 cases, ROSA 20–30 cases and NAVIO 18–28 cases. These differences may be attributed to system‐specific training protocols, user interface complexity and the degree of automation provided by each platform. Only in two studies by Probst et al. and Stelgelmann et al. were inexperienced surgeons considered. Interestingly, in these studies, the learning curve appeared longer, particularly in the study by Stelgelmann et al., where inexperienced surgeons required up to 50 cases to achieve proficiency. This highlights the critical role of prior surgical experience in shortening the adaptation phase to robotic systems. In two studies by Chen et al. and Vaidya et al., the surgeons’ level of experience was not specified. The number of surgeons whose level of experience is not known is specified only in the study by Chen et al., a total of 146 surgeons (74.5%, 146/193) (Table 3).

**Table 3:** Analysis.

First author	Number of cases	Surgeon's initial experience	Learning time	Assessment of learning time
Sodhi et al. [33]	240	Experienced	N/A	Reduction in operating time, no difference in operating time between the last 20 robotic cases and the last 20 traditional cases
Kayani et al. [13]	120 (60 Ra‐TKA)	Experienced	Six cases	Reduction in operating time
Kayani et al. [14]	120 (60 Ra‐TKA)	Experienced	Seven cases	Reduction in operating time, no differences in terms of limb alignment and implant positioning
Naziri et al. [22]	80 (40 Ra‐TKA)	Experienced	20 cases	Reduction in operating time
Marchand et al. [20]	200 (140 Ra‐TKA)	Experienced	N/S	Reduction in operating time. After 1 year, surgical time was shorter in Ra‐TKA compared to the traditional technique
Mahure et al. [19]	115	Experienced	10 cases	Reduction in operating time
Bell et al. [2]	60	Experienced	29 cases	Reduction in operating time
Savov et al. [31]	140 (70 Ra‐TKA)	Experienced	11 cases	Reduction in operating time. After 11 cases, the operating time for RA TKA was similar to that of the traditional technique
Probst et al. [27]	351	Not Experienced	Between 3 and 53 cases	Reduction in operating time with no significant differences in terms of accuracy (alignment), complications or patient satisfaction
Vermue et al. [40]	30	Experienced	Nine cases	Reduction in operating time with no significant differences in terms of accuracy (alignment) or complications.
Mei Lin Tay et al. [35]	101	Experienced	16 cases	Reduction in operating time of 10 min with no significant differences in terms of complications.
Morrisey et al. [21]	165 (66 Ra‐TKA)	Experienced	Two cases	Reduction of tourniquet time from 91 min (first two cases) to 65.63 min (cases 3‐10) to 63.48 min (last 40 cases), with no difference in complications.
Chen et al. [9]	N/A	N/A	12 cases	Reduction in operative time. Most of surgeons achieved the same operating times as traditional surgery by the 15th–20th case
Thiengwittayaporn et al. [36]	152 (75 Ra‐TKA)	Experienced	N/A	Reduction in operating time
Vanlommel et al. [38]	180 (90 Ra‐TKA)	Experienced	Between 6 and 11 cases, depending on the surgeon's experience	Reduction in operating time
Stegelmannet al. [34]	200	Not Experienced	50 cases	Average operating time of 121.1 min, with 3 revisions, 6 reoperations, 10 stiffness cases, 1 aseptic loosening, 1 superficial infection, 3 PJIs, and 1 refractory pain (first 100 cases), compared with 108.7 min, with 4 revisions, 9 reoperations, 9 stiffness cases, 1 aseptic loosening, 1 superficial infection, 0 PJIs, and 1 refractory pain (subsequent 100 cases)
Ali et al. [1]	120	Experienced	Between 1 and 20 cases, depending on the surgeon's experience	Reduction in operating time
Patel et al. [25]	604	Experienced	50 cases	Reduction in operating time
Vaidya et al. [39]	100 (75 Ra‐TKA)	N/A	25 cases	Reduction in operating time. After 25 cases, the operating time for RA TKA was similar to that of the traditional technique
Bolam et al. [5]	136 (53 Ra‐TKA)	Experienced	Between 5 and 15 cases, depending on the surgeon's experience	Reduction in operating time
Knenanidis et al. [15]	200	Experienced	70 cases	Reduction in operating time
Jung et al. [12]	100 (50 Ra‐TKA)	Experienced	18 cases	Reduction in operating time
Thongpulsawad et al. [37]	110	Experienced	Between 6 and 14 cases, depending on the surgeon's experience	Reduction in operating time of 22 min with no significant differences in terms of complications.
Weaver et al. [41]	500	N/A	Six cases	N/A
Neira et al. [23]	90	1 Experienced and 2 Not Experienced	Between 43 and 61 cases, depending on the surgeon's experience	Reduction in operating time from 91.9 min to 74.4 min for surgeons with less than 5 years of experience, and from 91.5 min to 72.7 min for experienced surgeons
Ejnisman et al. [11]	321	Experienced and Not Experienced	Between 30 and 40 cases, depending on the surgeon's experience	Reduction in operating time from 177.5 min (first 10 cases) to 145 min (last 10 cases) with no differences between initial and experienced surgeons
Zhang et al. [44]	90	Experienced	Between 8 and 16 cases, depending on the surgeon's experience	Reduction of average operating time from 112.67 min (average of the 30 cases) to 98.67 min (average of the last 10 cases).
Dragosloveanu et al. [10]	39	Experienced	Between three and six cases, depending on the surgeon's experience	Reduction of operating time from 115.4 min to 86.43 min, with no significant complications.

Studies comparing experienced and inexperienced surgeons, such as those by Ejnisman et al., Neira et al., and Stegelmann et al., indicate that experienced surgeons typically achieve proficiency within 15–30 cases, while inexperienced surgeons require 30–50 cases. In Stegelmann et al., two inexperienced surgeons needed 50 cases to reach stable operating times and outcomes. Similarly, Ejnisman et al. found that both experienced and inexperienced surgeons reached comparable proficiency after 30–40 cases, although the latter had significantly longer operating times in the early phase.

A difference in learning time is also observed between TKA and UKA. Studies such as Kayani et al. suggest that UKA has a shorter learning curve, with surgeons achieving proficiency in 6–10 cases, whereas TKA generally requires 15–30 cases. Variability exists depending on the robotic system used, with some platforms requiring more extensive adaptation.

## DISCUSSION

Robotic‐assisted total knee arthroplasty (Ra‐TKA) has revolutionised knee replacement surgery by introducing greater precision and control. However, its adoption necessitates overcoming a learning curve that varies based on the surgeon's experience. Operative times typically decrease as proficiency grows, and this improvement is independent of implant type, indicating that efficiency gains are primarily linked to mastering the robotic system.

Across studies, operative times consistently decrease as surgeons gain familiarity with robotic systems. For experienced surgeons, the learning curve is shorter and more predictable. Kayani et al. [13, 14] reported significant reductions in operative times after just 6–7 cases, with no compromise in alignment or implant positioning compared with traditional methods. Similarly, Bell et al. [2] found that 29 cases were required for experienced surgeons to equalise operative times between robotic and conventional approaches. Thiengwittayaporn et al. [36] and Vermue et al. [40] further observed that experienced surgeons stabilise their operative times within 9–11 cases, achieving reductions of up to 22 min per procedure. On average, experienced surgeons need approximately 15 cases to achieve consistent reductions in operative times.

In contrast, inexperienced surgeons require more cases to achieve proficiency. Probst et al. [27] highlighted a broad learning curve, with 3–53 cases needed for significant time reductions. Neira et al. [23] found that surgeons with less than 5 years of experience required 43–61 cases to reduce operative times from 91.9 min to 74.4 min. Similarly, Ejnisman et al. [11] reported operative times decreasing from 177.5 min in the first 10 cases to 145 min in the last 10, regardless of prior experience. On average, inexperienced surgeons need around 40 cases to achieve comparable time reductions, reflecting a steeper learning curve.

These findings underscore the importance of practice and adaptation in robotic surgery. Experienced surgeons achieve efficiency more quickly, but inexperienced surgeons eventually reach similar proficiency with adequate training and case volume. Probst et al. [27] highlighted that robotic systems enable less experienced surgeons to achieve outcomes comparable to their more experienced counterparts, making these systems valuable for training and education.

Despite the advantages of Ra‐TKA, significant barriers exist to its widespread adoption. One of the main challenges is the cost associated with robotic systems and surgeon training. The initial investment for acquiring robotic platforms, coupled with the expenses for specialised training programs, can be prohibitive for many healthcare institutions. In low‐resource settings, these costs may limit accessibility, preventing widespread implementation despite the clinical benefits. Additionally, limited availability of training centres and restricted access to robotic platforms can slow the learning process for surgeons interested in adopting these technologies. Expanding training opportunities through simulation‐based education, fellowships and structured mentorship programs could facilitate broader adoption.

Interestingly, the type of implant used during Ra‐TKA does not affect the learning curve. Patel et al. [25] and Zhang et al. [44] observed consistent reductions in operative times across different implant designs. This suggests that improvements in surgical efficiency are driven by familiarity with the robotic system rather than implant‐specific factors. These findings underline the versatility of robotic platforms in delivering consistent results regardless of the implant.

Comparing the learning curve of Ra‐TKA to other technological advancements in knee arthroplasty provides further insight into the adaptation process. Navigation‐assisted TKA, for instance, also introduced a learning curve when first implemented, with studies reporting that surgeons required approximately 20–30 cases to become proficient. However, unlike Ra‐TKA, navigation systems do not involve robotic arms or haptic feedback, making their workflows less complex. This suggests that while both technologies require an initial adaptation period, robotic surgery presents unique challenges due to its increased reliance on real‐time feedback and automated assistance. Understanding how surgeons adapted to previous technological advancements in arthroplasty could help refine training strategies for Ra‐TKA.

Complication rates are largely unaffected by the surgeon's experience after the initial learning phase. Thongpulsawad et al. and Zhang et al. [44] found no significant differences in alignment accuracy or patient safety despite reductions in operative times. Similarly, Probst et al. [27] reported no differences in alignment, patient satisfaction or complication rates between inexperienced and experienced surgeons. However, Stegelmann et al. [34] noted a higher incidence of stiffness and reoperations during the first 100 cases compared with later cases, indicating that early learning may pose slightly increased challenges.

To mitigate these risks during the learning phase, structured training programs and real‐time guidance from experienced mentors are essential. Implementing supervised proctoring for the first 15–20 cases may help inexperienced surgeons transition more smoothly to robotic techniques. Additionally, integrating simulation‐based training and stepwise exposure to robotic workflows before live surgical cases could enhance confidence and reduce intraoperative errors. The use of AI‐driven analytics to monitor surgeon performance and provide personalised feedback could further optimise the learning process, ensuring safer outcomes during early adoption.

While learning curve improvements are associated with reduced operative times and complication rates, the impact on functional recovery remains unclear. Future studies should incorporate PROMs (e.g., KOOS, WOMAC) to evaluate whether surgical proficiency translates to better patient outcomes.

The average number of cases needed to overcome these challenges aligns with the learning curve for operative time reductions. For experienced surgeons, 15 cases are typically sufficient to reach a plateau in both efficiency and complication rates. Inexperienced surgeons, however, often require around 40 cases to achieve comparable stability, as demonstrated by Neira et al. [23] and Ejnisman et al. [11]. Repetition and consistent exposure to robotic techniques are crucial to minimising risks during the learning phase.

The learning curve for Ra‐TKA differs from that of conventional TKA. In a multicenter study involving over 2000 patients, Whittaker et al. [43] examined the adoption of a new primary TKA implant system. They found that surgical times during the first 10 cases averaged 83.0 min, decreasing to 72.1 min with subsequent cases. The learning curve for conventional TKA is more influenced by implant‐specific technical requirements, whereas Ra‐TKA requires surgeons to master advanced technological workflows. Although both approaches show initial increases in surgical times, Ra‐TKA adds complexity through its reliance on technology.

While the learning curve is steeper for inexperienced surgeons, RA systems enable both groups to achieve comparable outcomes and complication rates with sufficient training and experience. Future efforts should focus on developing standardised robotic training protocols, expanding accessibility to robotic technology, and optimising learning strategies to facilitate smoother transitions for surgeons integrating Ra‐TKA into their practice.

## CONCLUSION

Reductions in operative times during Ra‐TKA are driven by surgeon familiarity with the robotic system and are independent of implant type. Experienced surgeons require fewer cases, approximately 15 (±15) to achieve consistent reductions in operative times and stable complication rates, while inexperienced surgeons may need around 40 cases (±19) to reach similar levels of proficiency. These findings underscore the importance of structured training programs and iterative practice to support surgeons in mastering robotic techniques. With appropriate preparation, robotic systems offer a pathway to standardised, efficient and safe surgical outcomes for all levels of experience.

## AUTHOR CONTRIBUTIONS

All authors have contributed to the conception and design of this study, acquisition of data, in drafting the article, in its revision, and all the authors approved the final draft of the submitted article. All authors have read and agreed to the published version of the manuscript.

## CONFLICT OF INTEREST STATEMENT

The authors declare no conflicts of interest.

## ETHICS STATEMENT

The authors have nothing to report. Written informed consent to publish this paper was not applicable. The informed consent was waived due to the anonymized retrospective design of the study.

## Data Availability

All the data we analysed and tables we compiled are available for any clarification.
